# Purpurin Suppresses *Candida albicans* Biofilm Formation and Hyphal Development

**DOI:** 10.1371/journal.pone.0050866

**Published:** 2012-11-30

**Authors:** Paul Wai-Kei Tsang, H. M. H. N. Bandara, Wing-Ping Fong

**Affiliations:** 1 Oral BioSciences, Faculty of Dentistry, The University of Hong Kong, Hong Kong, China; 2 Division of Pharmaceutics, College of Pharmacy, The University of Texas at Austin, Austin, Texas, United States of America; 3 School of Life Sciences, The Chinese University of Hong Kong, Hong Kong, China; David Geffen School of Medicine at University of California Los Angeles, United States of America

## Abstract

A striking and clinically relevant virulence trait of the human fungal pathogen *Candida albicans* is its ability to grow and switch reversibly among different morphological forms. Inhibition of yeast-to-hypha transition in *C*. *albicans* represents a new paradigm for antifungal intervention. We have previously demonstrated the novel antifungal activity of purpurin against *Candida* fungi. In this study, we extended our investigation by examining the *in* *vitro* effect of purpurin on *C*. *albicans* morphogenesis and biofilms. The susceptibility of *C*. *albicans* biofilms to purpurin was examined quantitatively by 2,3-bis(2-methoxy-4-nitro-5-sulfo-phenyl)-2H-tetrazolium-5-carboxanilide reduction assay. Hyphal formation and biofilm ultrastructure were examined qualitatively by scanning electron microscopy (SEM). Quantitative reverse transcription-PCR (qRT-PCR) was used to evaluate the expression of hypha-specific genes and hyphal regulator in purpurin-treated fungal cells. The results showed that, at sub-lethal concentration (3 µg/ml), purpurin blocked the yeast-to-hypha transition under hypha-inducing conditions. Purpurin also inhibited *C*. *albicans* biofilm formation and reduced the metabolic activity of mature biofilms in a concentration-dependent manner. SEM images showed that purpurin-treated *C*. *albicans* biofilms were scanty and exclusively consisted of aggregates of blastospores. qRT-PCR analyses indicated that purpurin downregulated the expression of hypha-specific genes (*ALS3*, *ECE1*, *HWP1*, *HYR1*) and the hyphal regulator *RAS1*. The data strongly suggested that purpurin suppressed *C*. *albicans* morphogenesis and caused distorted biofilm formation. By virtue of the ability to block these two virulence traits in *C*. *albicans*, purpurin may represent a potential candidate that deserves further investigations in the development of antifungal strategies against this notorious human fungal pathogen *in* *vivo*.

## Introduction

*Candida albicans* is a prevalent human fungal pathogen that poses significant medical challenge. It exists as a benign commensal in immunocompetent individuals but can become invasive and cause infections when the host immunity is impaired [1]. Recurrent lesions are not fatal; however, disseminated mycoses can be lethal with high morbidity and mortality (∼40–60%) [2]. In fact, candidiasis has been ranked fourth among leading types of nosocomial infections [3], [4]. The limited arsenal of conventional antifungal treatments for candidiasis relies heavily on polyenes, azoles and echinocandins. Unfortunately, they either have narrow therapeutic index, poor bioavailability, poor gastrointestinal absorption, or severe side effects [5], [6]. In addition, overuse of antifungal agents often leads to emergence of resistant strains that complicates the management of fungal infections in clinical settings [7].

The ability of *C*. *albicans* to colonize and proliferate in humans is closely related to its pathogenicity. One striking virulence trait of *C*. *albicans* is its ability to survive and switch reversibly between budded yeast and filamentous forms (pseudohyphae, true hyphae) [8], [9]. The phenotypic plasticity is tightly regulated by environmental cues. Cells grown in the presence of serum, or at high temperature (37°C), or under neutral/alkaline conditions, trigger hyphal formation [10]. The yeast-to-hypha transition is crucial for *C*. *albicans* infectivity. It is governed by the intracellular signalling pathways, for example, the cyclic AMP-protein kinase A (cAMP-PKA) and the Cek1 mitogen-activated protein kinase (MAPK) pathways which are tightly modulated by the membrane-bound GTPase (Ras1p) [11], [12]. Blockade of hyphal growth attenuates host’s tissue damage, and mutants defective in filamentation or locked in the yeast form are avirulent in systemic candidiasis [13], [14]. Hyphae not only help *C*. *albicans* to escape from host defence, but are also essential for pathogenicity by forming biofilms - heterogenous sessile communities of yeast and hyphal cells encased in extracellular matrix [15], [16]. *Candida* biofilms are highly resistant to standard antifungal treatments. Biofilms on various indwelling implanted devices, such as vascular/urinary catheters and denture, are excellent reservoirs to persistent fungal infections. It has been estimated that 80% of infections are biofilm-associated [17]. Therefore, impairment of hyphal development and biofilms may represent an effective and tangible measure to alleviate *C*. *albicans* pathogenesis, in line with the current antifungal paradigm that targets virulence traits instead of microbial eradication [18].

Purpurin (1,2,4-trihydroxy-9,10-anthraquinone) is a natural red anthraquinone pigment commonly found in madder root (*Rubia tinctorum* L.). It is an ingredient of herbal medicine and has been widely used as a food colouring agent [19]. We recently demonstrated the potent *in* *vitro* antifungal activity of purpurin against six *Candida* species [20]. Considering the repression of yeast-to-hypha transition attenuates *C*. *albicans* virulence/pathogenesis, the present study was designed to investigate the *in* *vitro* effect of purpurin on biofilms and hyphal development.

## Methods

### Strains, Cultivation and Chemicals

Wild type *C*. *albicans* strain SC5314 was routinely cultured in YPD agar (1% yeast extract, 2% peptone, 2% dextrose, 2% agar) at 30°C. To prepare a standard cell suspension, a single colony was inoculated into YNB medium (0.67% yeast nitrogen base w/o amino acids, 2% dextrose) and incubated for 18 h at 30°C with agitation. The fungal cells were harvested by centrifugation, washed twice in PBS (pH 7.2), and resuspended at 1×10^7^ cells/ml. Spider medium [21] and RPMI medium were used for hyphal induction at 37°C. Purpurin was purchased from TimTec Inc. (Newark, DE, USA). Stock solution (5 mg/ml) was prepared by dissolution in distilled dimethyl sulphoxide (DMSO) and kept at −20°C until use. The final concentration of DMSO was 1% in all assays. Other general chemicals were obtained from commercial suppliers with the highest grade available.

### Effect of Purpurin on *C*. *albicans* Biofilm Formation and Pre-formed Biofilms

Fungal biofilms were prepared as described [22] on commercially available, pre-sterilized, flat-bottomed 96-well polystyrene microtitre plates (Iwaki). Standard cell suspension of *C*. *albicans* (100 µl) was transferred into the wells and incubated for 1.5 h at 37°C with agitation. After the adhesion phase, the liquid was aspirated and each well was washed twice with PBS to remove loosely attached cells. Fresh YNB medium (200 µl) containing different concentrations (1 µg/ml to 10 µg/ml) of purpurin were added to each well and the plate was further incubated for 24 h at 37°C.

To investigate the effect of purpurin on pre-formed biofilms, *C*. *albicans* biofilms were prepared for 24 h at 37°C as described above. The wells were washed twice with PBS and fresh YNB medium (200 µl) containing different concentrations (1 µg/ml to 10 µg/ml) of purpurin were added and the plate was further incubated for 24 h at 37°C. YNB medium with 1% DMSO was included in control wells. The metabolic activity of the *C*. *albicans* biofilms was determined quantitatively using a standard 2,3-bis(2-methoxy-4-nitro-5-sulfo-phenyl)-2H-tetrazolium-5-carboxanilide (XTT) reduction assay.

### Quantitative Analysis - XTT Reduction Assay

At the end of the incubation, the supernatant was aspirated and the wells were washed twice with PBS. The fungal cell viability was determined using colorimetric XTT reduction assay that measures the activity of mitochondrial dehydrogenase [23]. XTT solution (1 mg/ml) was prepared by dissolving XTT powder in PBS, and the solution was filter-sterilized (0.22 µm pore size filter). XTT solution (40 µl) was mixed with freshly prepared menadione solution (0.4 mM; 2 µl) at 20∶1 (v/v) immediately prior to the assay. Thereafter, PBS (158 µl) was mixed with XTT-menadione solution (42 µl) and transferred to each well containing pre-washed biofilms, and incubated in the dark for 3 h at 37°C. After the incubation, the coloured supernatant (100 µl) was transferred to new microtiter plates, and the optical density of the supernatant was measured at 492 nm with a microtitre plate reader (SpectraMax 340 tunable microplate reader; Molecular Devices).

### Qualitative Analysis - Scanning Electron Microscopy

*C*. *albicans* biofilms were prepared on custom-made, tissue culture-treated, polystyrene coverslips as described [22]. Thereafter, the coverslips were washed twice with PBS and placed in 1% osmium tetroxide for 1 h. Samples were subsequently washed with distilled water, dehydrated in a series of ethanol solutions (70% for 10 min, 95% for 10 min and 100% for 20 min), and air-dried overnight in a desiccator prior to sputter coating with gold (JFC1 100; JEOL). The surface topographies of the *C*. *albicans* biofilms were viewed with a scanning electron microscope (Philip XL30CP).

### Effect of Purpurin on *C*. *albicans* Hyphal Growth on Solid Media

Standard cell suspension of *C*. *albicans* was spread on Spider plates or on YPD plates containing 10% fetal bovine serum (FBS) (Invitrogen) supplemented with DMSO or different concentrations (1 µg/ml to 5 µg/ml) of purpurin. The plates were incubated for 4 days at 37°C and the morphology of fungal colony was photographed using a digital camera.

### Effect of Purpurin on *C*. *albicans* Hyphal Growth in Liquid Media

Standard cell suspension of *C*. *albicans* was diluted (at 1×10^6^ cells/ml) in Spider medium or YPD medium containing 10% FBS or RPMI medium containing 10% FBS supplemented with DMSO or different concentrations (1 µg/ml to 5 µg/ml) of purpurin. Cells were grown at 37°C with agitation (250 rpm) for 4 h. Aliquots of fungal cells were visualized by differential interference contrast (DIC) microscopy and photographed.

### Evaluation of Purpurin Cytotoxicity

Primary human gingival fibroblasts (HGFs) were obtained from ScienCell Research Laboratories (Carlsbad, CA, USA). The cells were cultured in Dulbecco’s Modified Eagle’s Medium (DMEM) (Sigma) supplemented with 10% foetal bovine serum, penicillin (1 IU/ml), streptomycin (1 µg/ml) and L-glutamine (200 mM) in a humidified incubator at 37°C with 5% CO_2_ until 80% confluent. HGFs were subcultured by trypsinisation and cells of third passage were used. For cytotoxicity assay, HGFs (2×10^4^ cells; 100 µl) were seeded in 96-well microtitre plates for 24 h, followed by addition of different concentrations (0.1 µg/ml to 10 µg/ml) of purpurin in growth medium (100 µl). Cell viability was determined by 3-(4,5-dimethylthiazol-2-yl)-2,5-diphenyl-tetrazolium bromide (MTT) assay after 24 h. MTT solution (5 mg/ml) was prepared by dissolving MTT powder in PBS, and the solution was filter-sterilized (0.22 µm pore size filter) to remove insoluble residue. At the end of the incubation, the supernatant was aspirated and MTT solution (20 µl) was added to each well and incubated for 4 h at 37°C. The solution was replaced by DMSO (1 ml) to dissolve the dark blue formazan crystals. After incubation for 15 min at room temperature, the absorbance was read in microtitre plate reader at 570 nm.

### qRT-PCR Analysis of *C*. *albicans* Hypha-specific Genes and the Hyphal Regulator *RAS1*

Standard cell suspension of *C*. *albicans* (1 ml) was transferred into the wells of a pre-sterilized, flat-bottomed 24-well polystyrene microtiter plates (Iwaki). After incubation for 1.5 h at 37°C with agitation, the liquid was aspirated and each well was washed twice with PBS. Fresh YNB medium (1 ml) containing 3 µg/ml of purpurin was added to each well and the plate was further incubated for 24 h at 37°C. Control wells without the addition of purpurin were included for comparison. At the end of the incubation, the supernatant was aspirated and the wells were washed twice with PBS. Total RNA was extracted from *C*. *albicans* biofilms using SV Total RNA Isolation System (Promega) and 2 µg of template was reverse transcribed with Superscript II (Invitrogen). Resultant cDNAs were amplified by PCR and product specificity was confirmed by sequencing.

Real time PCR primers were designed for *C. albicans* hypha-specific genes (*ALS3*, *ECE1*, *HWP1*, *HYR1*), and the hyphal regulator *RAS1* using Primer Express Software Version 3 (Applied Biosystems) (Table 1). Primers were designed to generate amplicons with sizes between 50–150 bp and T_m_ between 58–60°C. Real time PCR mixture (20 µl) contained 10 µl of Fast SYBR Green Master Mix, 1 µl each of primers and 8 µl of sterile MilliQ water. The reactions were run on Step One Real time PCR Systems (Version 2, Applied Biosystems) (95°C incubation for 20 s, followed by 40 cycles of 95°C incubation for 1 s and 60°C for 20 s). Each primer pair produced a single amplicon with a uniform melting curve. A standard curve was constructed with a series of purified PCR products and the absolute copy number of amplicons was quantified (amplification efficiency >90%, R^2^>0.970). *EFB1* was used as a house keeping gene for reference (Table 1) [24]–[26].

**Table 1:** Primers used for qRT-PCR.

Primer	Sequence (5′–3′)
ALS3f	CTGGACCACCAGGAAACACT
ALS3r	ACCTGGAGGAGCAGTGAAAG
ECE1f	GTCGTCAGATTGCCAGAAATTG
ECE1r	CTTGGCATTTTCGATGGATTGT
HWP1f	GCCCAGAAAGTTCTGTTCCA
HWP1r	TTTGGTTTCAGTAGTAGTGGTTGG
HYR1f	TTGTTTGCGTCATCAAGACTTTG
HYR1r	GTCTTCATCAGCAGTAACACAACCA
RAS1f	CCCAACTATTGAGGATTCTTATCGTAAA
RAS1r	TCTCATGGCCAGATATTCTTCTTG
EFB1f	CATTGATGGTACTACTGCCAC
EFB1r	TTTACCGGCTGGCAAGTCTT

### Statistical Analysis

All experiments were performed in triplicate in three different occasions. All data were expressed as mean values with the corresponding standard deviations (SD). Differences between treated and control groups were assessed by Student’s *t* test or Mann-Whitney U test. In both tests, a *p*-value of <0.01 was considered statistically significant.

## Results

### Effect of Purpurin on *C*. *albicans* Biofilms

**Figure 1:**
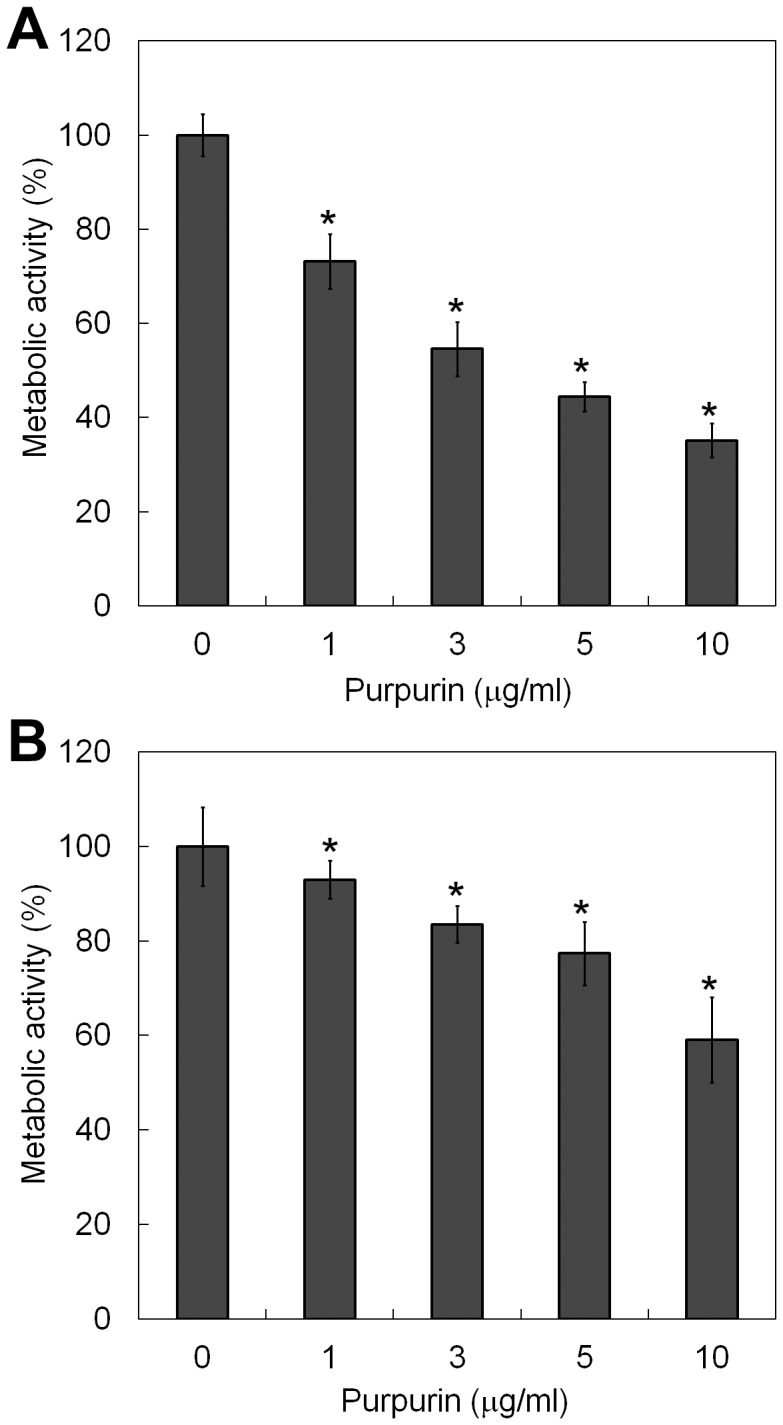
Effect of purpurin on *Candida albicans* biofilms. A) Biofilm formation of *C*. *albicans* SC5314 was examined in YNB medium containing the indicated concentrations of purpurin for 24 h at 37°C. B) Purpurin was added to pre-formed (mature) *C*. *albicans* biofilms and incubated in YNB medium for an additional 24 h at 37°C. The metabolic activity of the biofilms was assessed quantitatively using XTT reduction assay. The activity of samples without purpurin treatment (i.e. DMSO only) (0 µg/ml) was taken as 100%. Results shown were the average of three independent experiments ± SD. *p<0.01 when compared with the untreated controls.

**Figure 2:**
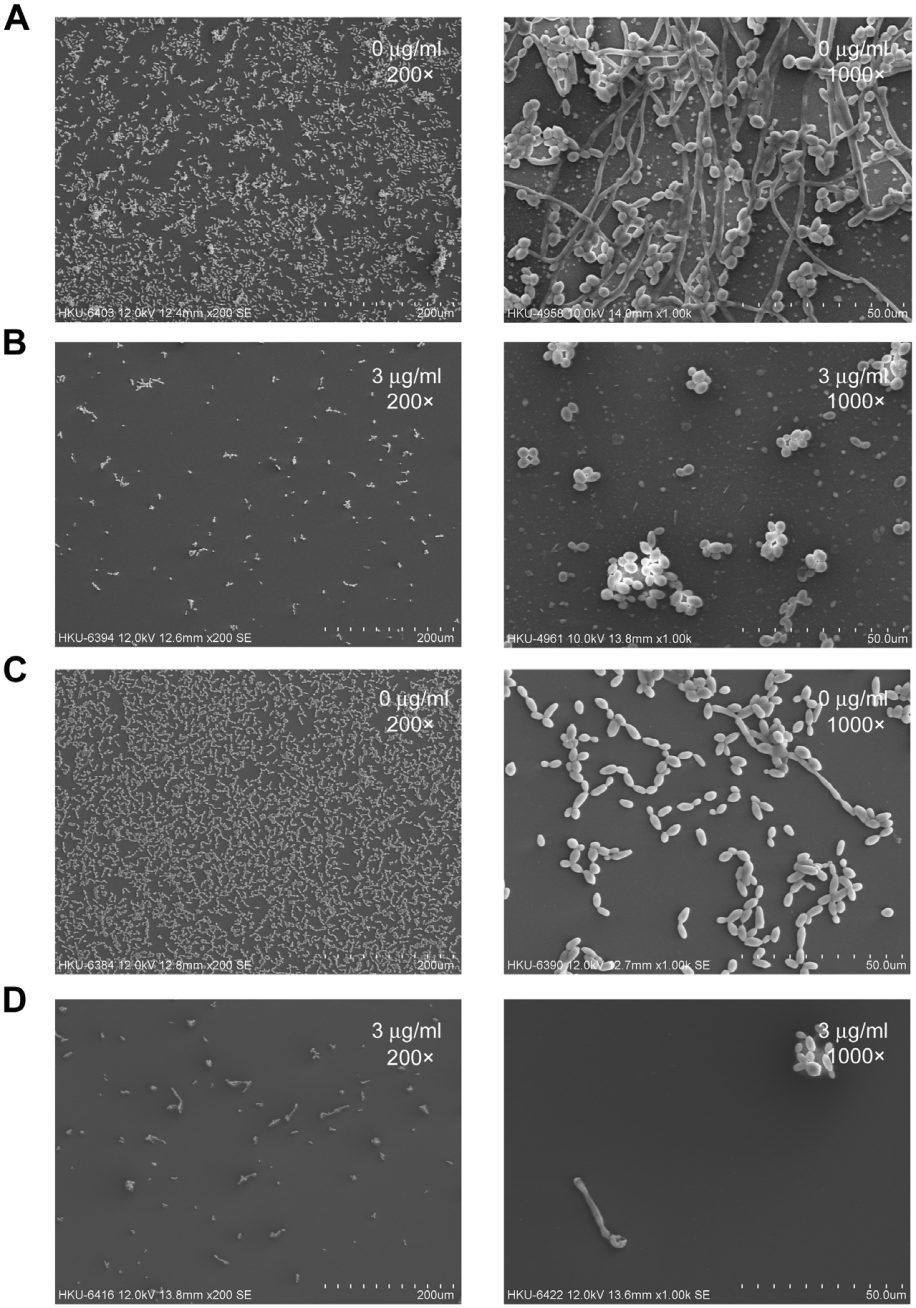
Scanning electron microscopy images of *Candida albicans* biofilms. Purpurin exhibited profound inhibitory effect on *C*. *albicans* biofilm formation and pre-formed biofilms. Fungal biofilms were formed on plastic coverslips, processed and coated with gold before viewing with a scanning electron microscope at low (200×) and high (1000×) magnifications. A) Biofilm formation without purpurin. B) Biofilm formation with 3 µg/ml of purpurin. C) Pre-formed biofilms without purpurin. D) Pre-formed biofilms with 3 µg/ml of purpurin.

The antifungal activity of purpurin on *C*. *albicans* biofilms was evaluated quantitatively using the XTT reduction assay. The effect was concentration-dependent, as reflected by a progressive reduction in cell viability, represented by metabolic activity, with increasing concentrations of purpurin (Fig. 1A). The metabolic activity of *C*. *albicans* during biofilm formation was reduced by ∼44% at 3 µg/ml and by ∼64% at 10 µg/ml. Pre-formed (mature) *C*. *albicans* biofilms were more resistant to the antifungal activity of purpurin, as revealed by a modest reduction in the cell viability by ∼6% at 3 µg/ml and by ∼37% at 10 µg/ml (Fig. 1B). SEM images showed that purpurin significantly suppressed *C*. *albicans* biofilm formation, *C*. *albicans* biofilms that were treated with purpurin were devoid of hyphal growth, scanty and exclusively consisted of aggregates of blastospores (Figs. 2A-D).

### Effect of Purpurin on *C*. *albicans* Hyphal Growth

**Figure 3:**
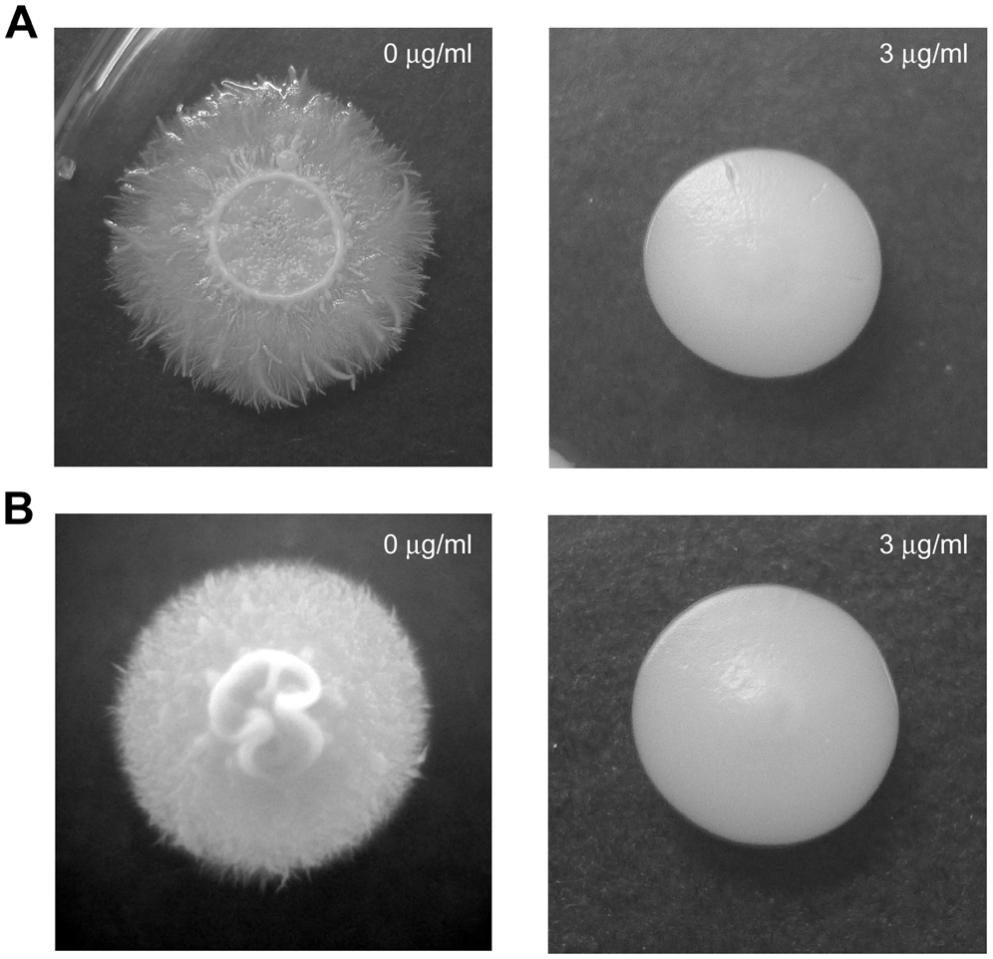
Effect of purpurin on hyphal formation in *Candida albicans*. *C*. *albicans* was spread on A) Spider agar or B) YPD agar containing 10% fetal bovine serum in the absence or presence (3 µg/ml) of purpurin. Morphology of fungal colony was photographed after incubation for 4 days at 37°C.

**Figure 4:**
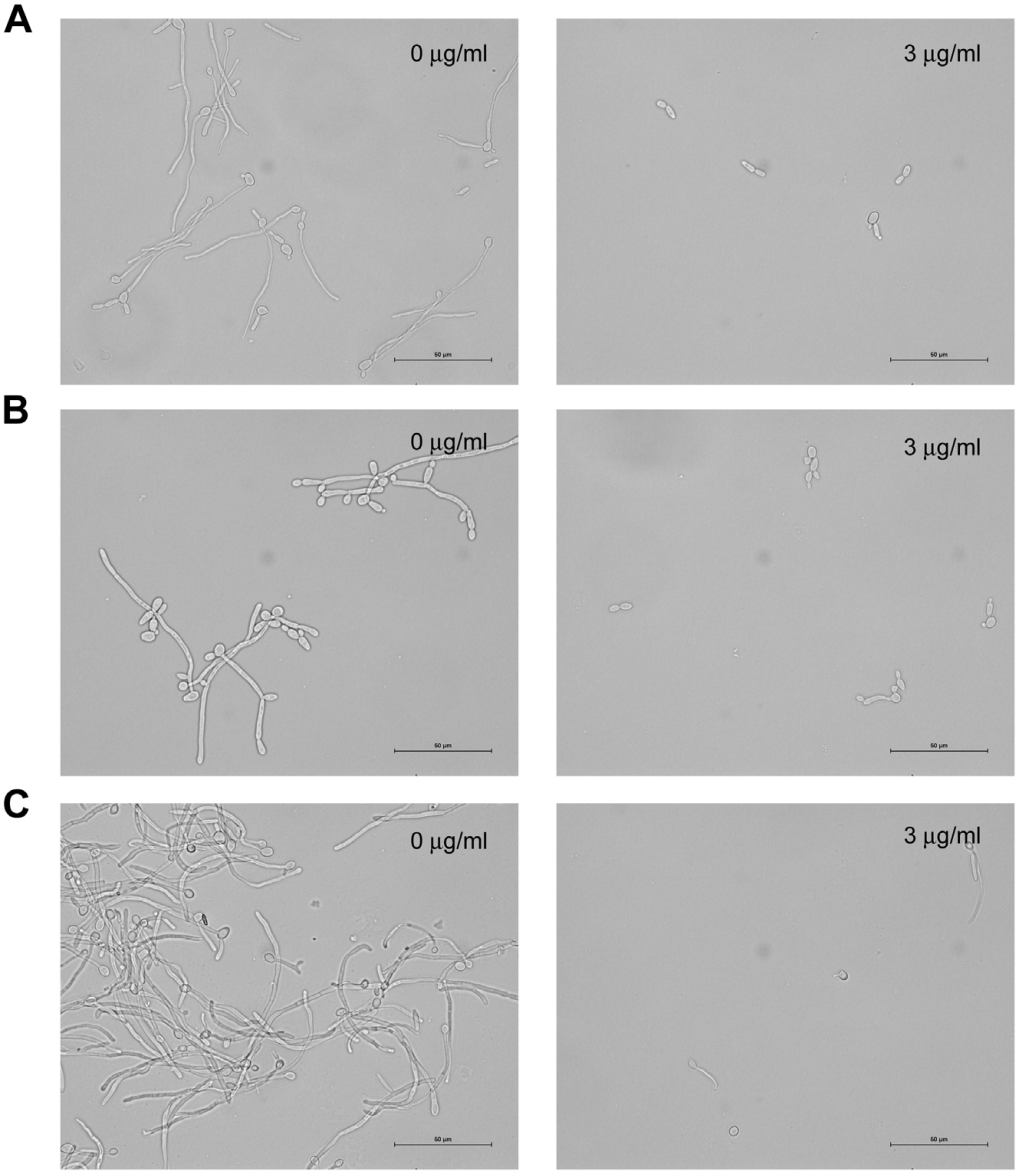
Effect of purpurin on hyphal growth of *Candida albicans* in liquid media. *C*. *albicans* was grown in A) Spider medium or B) YPD medium containing 10% FBS or C) RPMI medium containing 10% FBS in the absence or presence (3 µg/ml) of purpurin. Samples were withdrawn after incubation at 37°C for 4 h, and photographed at 100× magnification.

We examined the effect of purpurin on *C*. *albicans* hyphal growth on solid media by cultivating fungal cells and observing colony morphology on Spider agar at 37°C and on YPD agar containing 10% FBS (hypha-inducing conditions) supplemented with different concentrations of purpurin. It was found that 3 µg/ml of purpurin was sufficient to abrogate filamentation (Figs. 3A and B). Microscopic observation of the purpurin-treated fungal cells revealed an absence of filamentous cells whereas the control colony contained massive filamentation under both hypha-inducing conditions (data not shown). We also examined the effect of purpurin on hyphal growth in three different liquid media at 37°C. In both Spider medium and YPD medium containing 10% FBS, fungal cells grew as budding yeast form in the presence of 3 µg/ml of purpurin (Figs. 4A and B). However, in RPMI medium containing 10% FBS and 3 µg/ml of purpurin, some fungal cells grew as short pseudohyphae (Fig. 4C), and a higher concentration (5 µg/ml) of purpurin was required to complete inhibit filamentation (data not shown).

### Cytotoxicity of Purpurin

To evaluate whether purpurin is toxic to human cells, we used primary HGFs as a surrogate system. Purpurin was non-toxic to primary HGFs, the viability was 94% at 10 µg/ml as assessed by the MTT assay (data not shown).

### Effect of Purpurin on the Expression of Hypha-specific Genes and the Hyphal Regulator *RAS1*

**Figure 5:**
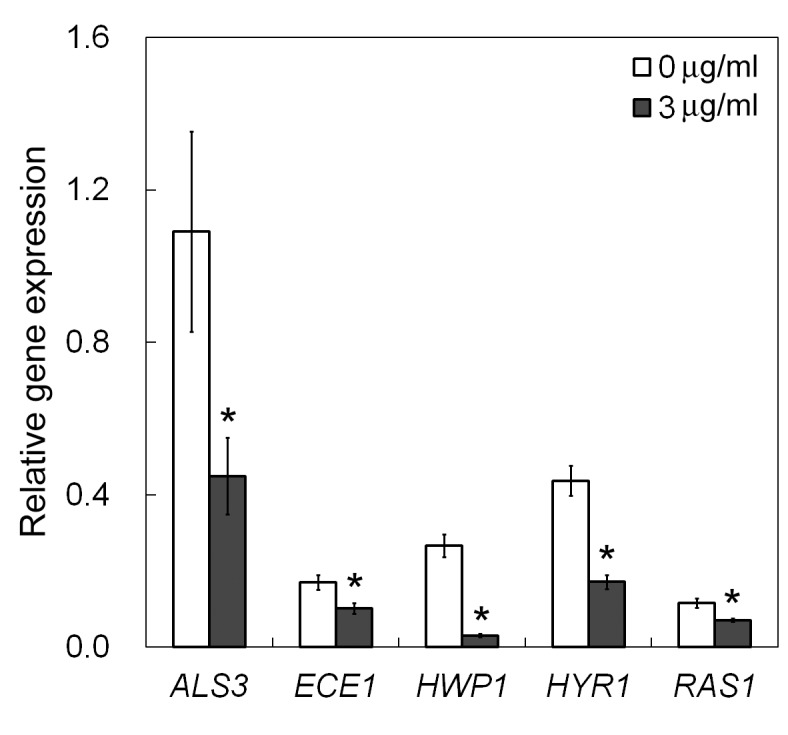
qRT-PCR analysis of expression of hypha-specific genes and the hyphal regulator *RAS1*. *Candida albicans* cells were incubated in the absence or the presence (3 µg/ml) of purpurin, and the expression of the target genes were determined by qRT-PCR. Housekeeping gene *EFB1* was used for normalization. Results shown were the average of three independent experiments ± SD. *p<0.01 when compared with the respective controls.

To uncover the underlying relationship between purpurin and *C*. *albicans* morphogenesis, we performed qRT-PCR to assess the expression of hypha-specific genes and the hyphal regulator *RAS1* in *C*. *albicans* with purpurin. Purpurin downregulated the expression of the hypha-specific genes, albeit to various extents (Fig. 5). In particular, when compared with their respective untreated controls, the expression of *HWP1* was reduced by >88%; while the transcript levels of *ALS3*, *HYR1* and *ECE1* were lowered by ∼59%, ∼61%, and ∼40% respectively. The expression of the hyphal regulator *RAS1* was reduced by almost 40%.

## Discussion

Invasive fungal infections have long been serious problems to human health, especially to the immunocompromised population. Despite years of effort, the battle against candidiasis has remained largely unsuccessful. In view of the limited efficacy of the current antifungal treatments and the emergence of multidrug resistant *Candida* strains, it is in dire need of novel antifungal approaches. Plant materials have been regarded as ample sources of novel biomolecules with a broad spectrum of biological and pharmacological properties. Bioactive compounds with antimicrobial activity have been identified [27]; many of them are potent against diverse fungal pathogens [28].

In an earlier study, our laboratory reported that purpurin exhibited potent antifungal activity against *C*. *albicans* with a minimum inhibitory concentration of 5.12 µg/ml, and concluded that the antifungal mechanism of purpurin might involve perturbation of mitochondrial homeostasis that triggers cellular apoptosis [20]. In the present study, we extended our investigation of purpurin on *Candida* morphogenesis and biofilms using both qualitative and quantitative methods. SEM was employed as a qualitative analytical tool to reveal the morphology and architecture of *Candida* biofilms at high magnification. The efficacy of purpurin on *Candida* biofilms was evaluated quantitatively using XTT reduction assay. Several methods are available to quantitatively assess the viability of *C*. *albicans* biofilms, and a recent study clearly demonstrated that XTT reduction assay provided the most reproducible and accurate measurement [29]. Inhibition on yeast-to-hypha transition and biofilms was concentration-dependent. Three µg/ml of purpurin was sufficient to completely inhibit filamentation under most hypha-inducing conditions in both solid and liquid media, except a slightly higher concentration of purpurin (5 µg/ml) was needed to completely inhibit hyphal growth in RPMI medium containing 10% FBS. The discrepancy in the inhibitory effect of purpurin on hyphal growth under different hypha-inducing conditions might be due to the complex nature of filamentation in *C*. *albicans* in response to environmental signals, or to other unknown molecular interactions in the presence of purpurin. A similar phenomenon of medium-dependent inhibition of filamentation in *C*. *albicans* was also observed in the presence of conjugated linoleic acid [30]. Mature biofilms of *C*. *albicans* were less susceptible to purpurin, probably because the well-established biofilm architecture resists the penetration of purpurin.

Numerous studies have been performed to identify small molecules that modulate hyphal formation [31]–[33]. Hyphae are integral components in biofilms whose formation has profound effect on human health and imposes medical challenges. Dentures and intravenous catheters for medical treatments are substrates for biofilm development [34]. Formation of biofilms is a step-wise process. Yeast cells first adhere to the substratum, followed by initiation of hyphal growth. More extracellular matrix is accumulated in the maturation step and the biofilm structure is formed. Finally, yeast cells detach and invade surrounding tissues [35]. Respective hypha-specific genes and regulatory pathways have been identified in different stages of biofilm development. To gain insight into the effect of purpurin on *C*. *albicans* morphogenesis, we measured the expression of four hypha-specific genes, viz. *ALS3*, *ECE1*, *HWP1*, *HYR1*, under hypha-inducing conditions. These genes encode proteins that are essential for cell wall integrity and play role in the initiation step of hyphal development. Agglutinin-like sequence 3 (*ALS3*) and hyphal wall protein 1 (*HWP1*) are cell wall proteins that play key role in intercellular adherence and cell-substrate interactions for proper formation of three-dimensional biofilm architecture [36], [37]. *HYR1* encodes a cell surface glycosylphosphatidylinositol-anchored protein which confers *C*. *albicans* resistance to phagocyte-mediated killing [38], [39], and also itself a potential target for vaccine development [40]. *ECE1* is essential for cell elongation and biofilm formation [41]. Our data on transcript analysis showed that their expression was reduced, and therefore purpurin might exert its antibiofilm effect via perturbation of cell wall integrity. Hyphal development in *C*. *albicans* is tightly controlled by regulatory circuits that respond to environmental cues. In *RAS1*-mediated way, the membrane localized GTPase Ras1p activates the cAMP-PKA and the Cek1-MAPK pathways to enhance the levels of Efg1p and Cph1p respectively for hyphal growth [12]. Expression of the hyphal regulator *RAS1* was downregulated in *C*. *albicans* in the presence of purpurin, implying that purpurin might inhibit the expression of hypha-specific genes via an *RAS1*-dependent manner. The physiological role of *RAS1* in *C*. *albicans* in response to purpurin can be assessed by Ras1p overexpression that warrants further investigation.

*C*. *albicans* poses a serious health threat to humans and puts heavy economic burden on our society. Instead of cell killing, blocking of virulence traits of pathogens has been recently considered as a new antifungal paradigm [18]. It is thus conceivable that suppression of the yeast-to-hypha transition in *C*. *albicans* by purpurin could be a viable antifungal strategy, with the advantage of a reduced likelihood of acquiring drug resistance owing to the lower effective concentrations at sub-lethal levels.

In conclusion, the present study demonstrated the *in* *vitro* effect of purpurin on *C*. *albicans* hyphal formation and biofilm development. Purpurin lowered the expression of *RAS1* and hypha-specific genes in *C*. *albicans*. In addition, the non-toxic nature of purpurin to human cells may have clinical relevance as a new method to treat candidiasis. Experiments on infection models with indwelling catheters are being carried out to evaluate the *in* *vivo* application of purpurin.
